# Recruitment of Participants and Delivery of Online Mental Health Resources for Depressed Individuals Using Tumblr: Pilot Randomized Control Trial

**DOI:** 10.2196/resprot.9421

**Published:** 2018-04-12

**Authors:** Erin Kelleher, Megan Moreno, Megan Pumper Wilt

**Affiliations:** ^1^ Seattle Children's Research Institute Center for Child Health, Behavior and Development Seattle, WA United States; ^2^ University of Washington Seattle, WA United States

**Keywords:** adolescents, depression, intervention, social media, tumblr

## Abstract

**Background:**

Adolescents and young adults frequently post depression symptom references on social media; previous studies show positive associations between depression posts and self-reported depression symptoms. Depression is common among young people and this population often experiences many barriers to mental health care. Thus, social media may be a new resource to identify, recruit, and intervene with young people at risk for depression.

**Objective:**

The purpose of this pilot study was to test a social media intervention on Tumblr. We used social media to identify and recruit participants and to deliver the intervention of online depression resources.

**Methods:**

This randomized pilot intervention identified Tumblr users age 15-23 who posted about depression using the search term “#depress”. Eligible participants were recruited via Tumblr messages; consented participants completed depression surveys and were then randomized to an intervention of online mental health resources delivered via a Tumblr message, while control participants did not receive resources. Postintervention online surveys assessed resource access and usefulness and control groups were asked whether they would have liked to receive resources. Analyses included *t* tests.

**Results:**

A total of 25 participants met eligibility criteria. The mean age of the participants was 17.5 (SD 1.9) and 65% were female with average score on the Patient Health Questionnaire-9 of 17.5 (SD 5.9). Among the 11 intervention participants, 36% (4/11) reported accessing intervention resources and 64% (7/11) felt the intervention was acceptable. Among the 14 control participants, only 29% (4/14) of reported that receiving resources online would be acceptable (*P*=.02). Participants suggested anonymity and ease of use as important characteristics in an online depression resource.

**Conclusions:**

The intervention was appropriately targeted to young people at risk for depression, and recruitment via Tumblr was feasible. Most participants in the intervention group felt the social media approach was acceptable, and about a third utilized the online resources. Participants who had not experienced the intervention were less likely to find it acceptable. Future studies should explore this approach in larger samples. Social media may be an appropriate platform for online depression interventions for young people.

## Introduction

Depression is among the most common illnesses affecting adolescents and young adults but many do not seek clinical care or know about available resources [1-3]. Previous studies have illustrated several barriers to mental health care among young people, including recognition of a problem, access to care, fear of stigma if care is accessed, and identification of young people–specific resources [4-6]. Many of the services offered do not address the needs of young people during the later years of adolescence and young adulthood [4]. Given these barriers to mental health care, innovative solutions are needed. Online approaches may be one such solution.

Due to the continuous rise in social media use among young people, researchers are investigating the impact and influence of online mental health resources [7,8] *.* The vast majority of young people are online and most of these young people use social media to express themselves and get social support [9-11]. Many social media users report feeling more comfortable self-disclosing information online rather than in person [11]. Previous studies suggest depression symptom disclosures on social media are common and are associated with offline depression symptoms [12,13]. One study found one-third of public college Facebook profiles displayed at least one reference to a depression symptom and 2.5% of Facebook profiles fit the Diagnostic and Statistical Manual (DSM-IV) criteria for a major depressive episode [13]. Thus, it is possible that social media posts could be used to identify young people at risk for depression based on posting references to depression symptoms. In this way, young people could be identified and even provided resources or support online.

There are several ways in which online and social media interventions present opportunities to reach and engage with young people [9,14,15]. Online and social media interventions often cost less and allow researchers to reach a more diverse population [14,15] **.** Reaching young people is a critical challenge as young people are the least likely age group to have a primary care provider and a majority do not seek professional help for mental health problems [2,6,16]. Previous online intervention studies had more participation from underrepresented and underserved individuals compared to traditional intervention methods [14,17]. Online interventions allow individuals to access the intervention anytime without geographical and time barriers. A majority of the successful online intervention studies used structured online programs that included education and coping strategies as their means of intervention [3,8,18-21]. Many young people prefer online intervention options because they offer privacy, anonymity, 24-hour accessibility, and avoid face-to-face interactions [9,22].

While previous online interventions have addressed alcohol use, sexual behaviors, depression, and other mental health disorders, none of those interventions offered online resources for adolescents to use independently and a majority of studies did not use social media for participant identification and recruitment [3,21,23]. The purpose of this study was to determine whether a social media intervention offering resources to young people displaying references to depression appropriately targeted young people with depression and was accessed by, and deemed acceptable by, young people.

## Methods

### Study Setting

This randomized control pilot intervention occurred from October 2014 to December 2015 on Tumblr [24]. Tumblr is a popular social media site among young people that allows profiles to be created and displayed anonymously [7]. An email address and username is the only information Tumblr requires to create an account. Tumblr users are not required to display identifying information on their profile page. The email address provided to create a profile is not visible to other users. Thus, users can post personal multimedia content labeled with hashtags with fewer restrictions compared to Facebook or Twitter. Similar to Facebook and Twitter, users can repost, like, and comment on other user’s posts. A majority of Tumblr blogs are publically available and profiles and posts can be viewed without having a Tumblr profile [25]. The Western Institutional Review Board approved this pilot study.

### Participants

The goal for this pilot study was to identify participants who displayed depression symptoms on social media. The target sample size was 45 for this pilot test. Potential participants were identified via a post on Tumblr using the search term #depress to encompass both key words “depression” and “depressed,” and search filter of “most recent posts.”

A codebook from a previous study was used to determine whether the post displayed a reference to depression [12]. The codebook was created using the DSM-IV diagnostic criteria for a major depressive episode (see Table 1). Examples of posts eligible for participation included: “I feel like I am not good enough for anyone #depress #sad;” “I can’t do anything right. Straight A’s still isn’t good enough for my parents. I’m so exhausted and I am hurting physically and emotionally. #depress #zzz #alone #depression”; and a picture of cuts on an arm with the caption, “they thought I was fine. #alone #depress #tears #die”.

After a post was determined to meet the depression codebook criteria, the profile of the individual that created the post was reviewed. Participants whose Tumblr profiles were in English, displayed their profile age as between 15 and 23 years, and displayed one or more depression symptoms consistent with the DSM-IV in their Tumblr post within the last 14 days were considered eligible [13,26].

Individuals with Tumblr profiles who displayed other mental health comorbidities (ie, #bipolar) were excluded. Additional inclusion criteria were the ability to receive private Tumblr messages and presence of timestamps on profile posts.

**Table 1:** Depression codebook criteria.

Category	Terms or phrases to include	Terms or phrases to exclude
Depressed mood	Sad, empty, crying, tearful, alone, lonely, sad face emoticon	“I had a bad day”, “FML”
Decreased interest or pleasure in activities	Not having fun, don't feel like doing anything, giving up	
Increase/decrease in appetite	No appetite, don't feel like eating, can't stop eating, eating everything in sight	“I ate too much at McDonalds this weekend,” references to poor eating habits rather than changes in appetite
Sleep problems	Sleeping too much, slept more than 10 hours, fatigue, tired, exhausted	
Psychomotor agitation/retardation	Feeling slow	
Loss of energy	Can't get anything done, no motivation	
Feelings of guilt, worthlessness, negative self-appraisal	Feels guilty or worthless, “I'm so stupid”	
Indecisiveness	Can't decide on something, don't feel like deciding, can't make up mind	
Recurrent thoughts of death or suicidal ideation	Thinking of ways to commit suicide, references to jumping	
Difficulty concentrating	Can't study, can't finish work, can't concentrate	Don't want to concentrate, can't concentrate because of activity (TV, friends, Facebook)

### Intervention

The intervention tested in this research protocol was designed to be delivered online and provide online depression resources. A resource sheet was developed in consultation with an adolescent health mental health expert listing mental health resources that were nationally available, free, online, and publicly accessible (Multimedia Appendix 1). Not all of the resources provided were explicitly for young people, however some of the resources had specific pages or topics specifically for young people. Because a majority of young people do not seek help or know how to access mental health resources, the resource sheet offered a variety of depression resources for participants to access [1]. The resources included chat rooms, information-based websites, hotlines, and means to find a mental health professional for depression. A majority of the resources could be accessed 24 hours a day. The resource sheet was delivered using a study team Tumblr profile via a private Tumblr message explaining the study and the research team (Multimedia Appendix 1). Participants were asked to message the research group via Tumblr if they did not wish to be contacted again by the research group.

### Procedures

**Figure 1:**
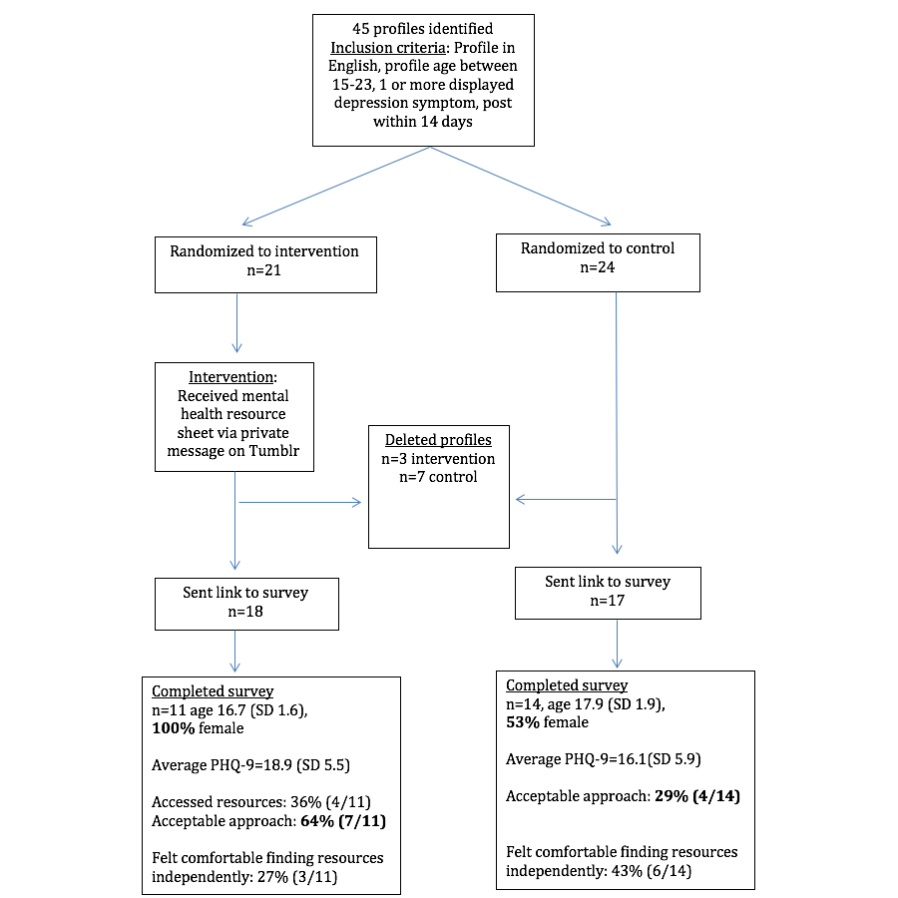
Flowchart of study design. *P* values calculated using an unpaired *t* test. Bolded values indicate significant values. PHQ-9: Patient Health Questionnaire-9.

After eligible individuals’ Tumblr profiles were identified and randomized, the username, profile URL, post with the depression symptom reference, and hashtags of participants were stored in an excel spreadsheet. Participants were randomized using a randomization website [27]. The numbers 1 through 45 were randomized into intervention and control numbers. Participants were assigned numbers in the order their post appeared on Tumblr. Intervention participants received a resource list as the intervention while the participants in the control group were not initially contacted. One month after the intervention was delivered, a private Tumblr message was sent to both groups that included a link to the secure, anonymous online survey and consent form. Participants were provided information about the research group and how to contact the group, anonymity of the survey, how long the data would be stored, and how to exit the survey. There was one question per page with a maximum of 22 questions depending on how the participant answered specific questions. Participants were not required to provide an answer for any of the questions and were provided a back button to change previous answers. Due to the anonymity of the survey, researchers were not able to identify which participants completed the survey. IP addresses were verified to prevent duplicate entries from the same user. Intervention procedures are described in Figure 1. Participants who completed the survey were provided a $10 gift card.

### Variables

#### Feasibility of Recruitment

In order to assess the feasibility of recruiting teens at risk for depression, our survey included the Patient Health Questionnaire-9 (PHQ-9) [28]. The PHQ-9 is a 9-item measure that assesses DSM-IV criteria for depression within the last two weeks and is commonly used as a clinical screening tool across the United States with the following Likert scaled responses (0=Not at all, 1=Several days, 2=More than half days, and 3=Nearly every day). The scores on the PHQ-9 were computed and categories of depression level were established based on the PHQ-9 scoring guidelines: none to minimal depression (0-4 score), mild depression (5-9 score), moderate depression (10-14 score), moderate to severe depression (15-19 score), and severe depression (20-27 score). We hypothesized that if the average PHQ-9 score was in the range of mild to moderate depression, our search strategy was a feasible method of identifying teens at-risk for depression.

#### Access to intervention resources

To assess whether intervention participants accessed resources, we asked the intervention group on the follow-up survey to describe resources the participant accessed or viewed and which ones they found helpful. To understand participants’ access to online mental health resources in general, all participants were asked whether they knew of, felt comfortable accessing, or used other online depression resources in the past.

#### Acceptability of intervention approach

For both groups, the survey assessed acceptability of the approach of sending resources via Tumblr. The control group survey asked if they would have liked to receive depression resources and if the intervention approach would be acceptable.

#### Participant suggestions

All participants were asked to provide suggestions on key components of online mental health interventions that would support their acceptability.

### Analysis

Descriptive data were summarized; group comparisons and comparison of PHQ-9 scores postintervention were both conducted using an unpaired *t* test. There was no blinding for this study.

## Results

### Participants

Among the 45 participants initially selected, 21 were randomized to the intervention. A total of 10 (3 intervention) participants deleted their profiles before receiving a survey. There were 25 completed surveys, 65% (16/25) were female and were an average age of 17.5 years (SD 1.9; Figure 1). The average PHQ-9 score was 17.5 (SD 5.4), not differing among groups. None of the participants messaged the researchers to not be contacted again.

### Intervention Group

Among intervention participants (n=11), 64% (7/11) of the participants found this intervention method to be an acceptable approach. Roughly one-third (4/11) of participants accessed intervention resources. Of those 4 participants, 100% (4) felt the approach was acceptable and 75% (3/4) answered they would likely use the resources again. The Depression Chat Rooms, IM ALIVE, and National Suicide Hotline were the resources accessed. Only 27% (3/11) felt comfortable finding resources independently. Before receiving the resources, half of the participants that accessed the resources did not know of other online resources for mental health (2/4).

### Control Group

Among control group participants (n=14), only 14% (2/14) of the control group would have wanted to receive resources online and 29% (4/14) of control group participants reported they thought the intervention would be an acceptable approach. There was a statistically significant difference between the acceptability of the intervention approach between the intervention and control groups (*P*=.02). Although 71% (10/14) of control participants had used online resources for mental health, only 43% (6/14) felt comfortable finding resources independently. Common resources accessed by control participants included WedMD, psychcentral, anonymous chat groups and hotlines, and Tumblr.

### Participant Suggestions

Overall, participants in both groups felt anonymity, supportiveness, and ease of use as important characteristics in an online depression resource. Participants in the control group found websites with young people going through similar experiences and chat groups to be the most helpful resources. A majority (9/11) of participants in the intervention group were not sure or did not have resources to suggest. The two suggestions were to offer cognitive behavior therapy resources and www.emotionalbaggage.com.

## Discussion

### Principal Findings

The research protocol tested in this pilot intervention study included an online mental health resource sheet and used Tumblr to identify teens that posted about depression as well as to deliver the resource. We found that the intervention was appropriately targeted to young people at risk for depression and recruitment via Tumblr was feasible.

The young people in this study had an average PHQ-9 score of 17.5, which can be interpreted as moderately severe depression [28]. Thus, identifying participants based on depression posts on Tumblr targeted the appropriate population for this study. Findings are similar to previous work in which depression displays on Facebook were associated with self-reported depression symptoms [13]. Therefore, displayed depression references on Tumblr could be a helpful tool in identifying young people at risk for depression.

Over one-third of participants in the intervention group accessed the online resources provided. The participants that used the resources found the resources to be helpful and stated they would use them in the future. Participants utilized the anonymous chat rooms and hotlines, which were similar to many of the resources accessed independently by the control group. However, it is important to highlight that Tumblr and WebMD, though unverified by a mental health expert, were considered depression resources for some participants in the control group. It is possible that participants in the control group did not find this approach acceptable because they were unaware of different kinds of available online mental health resources and therefore did not think online resources could be helpful.

It is notable that more than half of control participants did not feel comfortable finding online mental health resources independently. However, they suggested and accessed similar online depression resources the intervention group received. A majority of participants in both groups found anonymity, supportiveness, and accessibility to be helpful characteristics in an online mental health resource. These findings are similar to previous online intervention studies for young people [9,22]. Of importance, the intervention resource list encompassed the characteristics mentioned above.

### Limitations

There were several limitations to the study. Only one social media site was used and the sample size was small, therefore findings cannot be generalized; however, this study was intended to test a research protocol and determine whether it could be used in future studies. Due to profile deletion and nonresponse to the survey, the intervention group had only female participants. However, males and females are equally likely to post a reference to depression on social media [12,29]. A previous depression intervention study did not find a statistically significant difference in the success of the intervention between males and females [3]. The deletion of several profiles in the course of this study was unexpected, further study could investigate whether other social media sites promote more stable or long-lasting profiles. Due to the anonymity of the surveys and the priority to protect participant identities, researchers had no capacity to follow up if participants answered that they had thoughts self-harm or suicide. The anonymity of the survey also prevented researchers from contacting participants to remind them to take the survey. This could be an explanation for the low percentage of completed surveys.

### Future Studies

Findings support future work to test this approach and should consider the following:

- Obtain larger sample sizes with less restrictive inclusion criteria. Many profiles were not able to be included due to the age, language, and privacy restrictions.
- Utilize other social media sites such as Facebook or Twitter to reach a larger population of social media users.
- Follow up with participants for feedback on why they found the intervention and certain resources to be helpful or not helpful and whether this approach was considered intrusive. This would allow a mental health expert to verify a resource list more suitable for young people.

### Conclusions

Previous successful online intervention studies have not incorporated social media for the identification and recruitment of participants or to offer mental health resources. Findings support the need for mental health resources targeting the adolescent and young adult population, and this study offers a novel approach for offering these services. Social media is an appropriate platform for mental health interventions for young people because it can reach a diverse population while remaining cost effective. This intervention protocol could be utilized and expanded in future studies to further understand resources utilized by young people and find new approaches to reaching this population.

## Appendix

Multimedia Appendix 1 Resources sent to intervention participants.

Multimedia Appendix 2 CONSORT-EHEALTH checklist (V 1.6.1).

## Abbreviations

DSM-IV: Diagnostic and Statistical Manual
PHQ-9: Patient Health Questionnaire-9
