# Supplementary figures and images for: Recruitment of Participants and Delivery of Online Mental Health Resources for Depressed Individuals Using Tumblr: Pilot Randomized Control Trial
Source: JMIR Res Protoc. 2018 Apr 12;7(4):e95. doi: 10.2196/resprot.9421 (PMC5920159; doi:10.2196/resprot.9421)

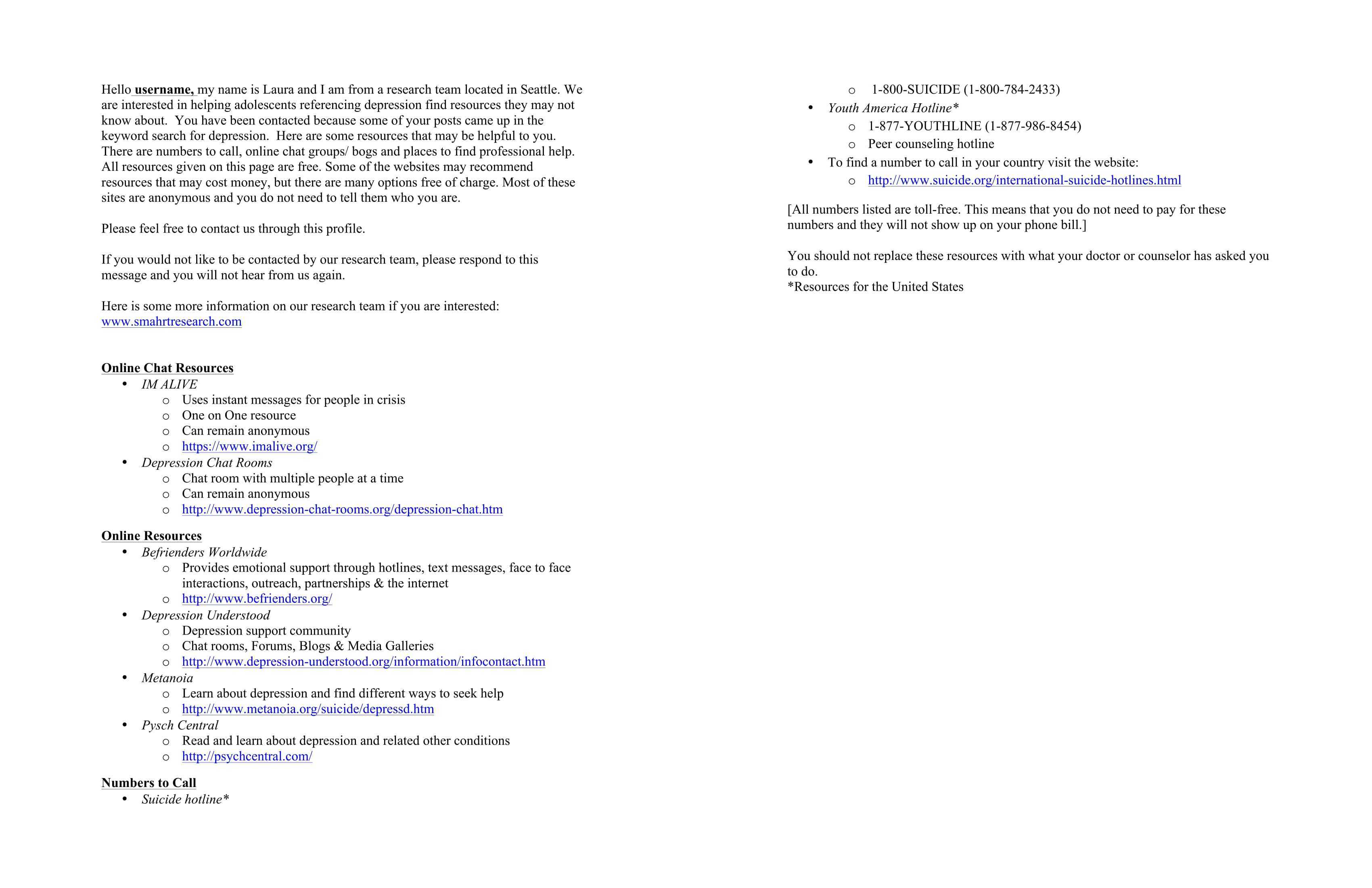

Supplement: Multimedia Appendix 1 [file resprot_v7i4e95_app1.jpg]
